# Nanoconfined Water Clusters in Zinc White Oil Paint

**DOI:** 10.1021/acs.jpcc.3c04720

**Published:** 2023-09-15

**Authors:** Jorien R. Duivenvoorden, Federico Caporaletti, Sander Woutersen, Katrien Keune, Joen J. Hermans

**Affiliations:** † Van ‘t Hoff Institute for Molecular Sciences, University of Amsterdam Science Park 904, 1098 XH Amsterdam, The Netherlands; ‡ Conservation & Science, Rijksmuseum Hobbemastraat 22, 1071 ZC Amsterdam, The Netherlands; § Laboratory of Polymer and Soft Matter Dynamics, Experimental Soft Matter and Thermal Physics, Université Libre de Bruxelles Avenue, Franklin Roosevelt 50, 1050 Brussels, Belgium; ∥ Conservation & Restoration, Amsterdam School of Heritage, Memory and Material Culture, University of Amsterdam Turfdraagsterpad 15-17, 1012 XT Amsterdam, The Netherlands

## Abstract

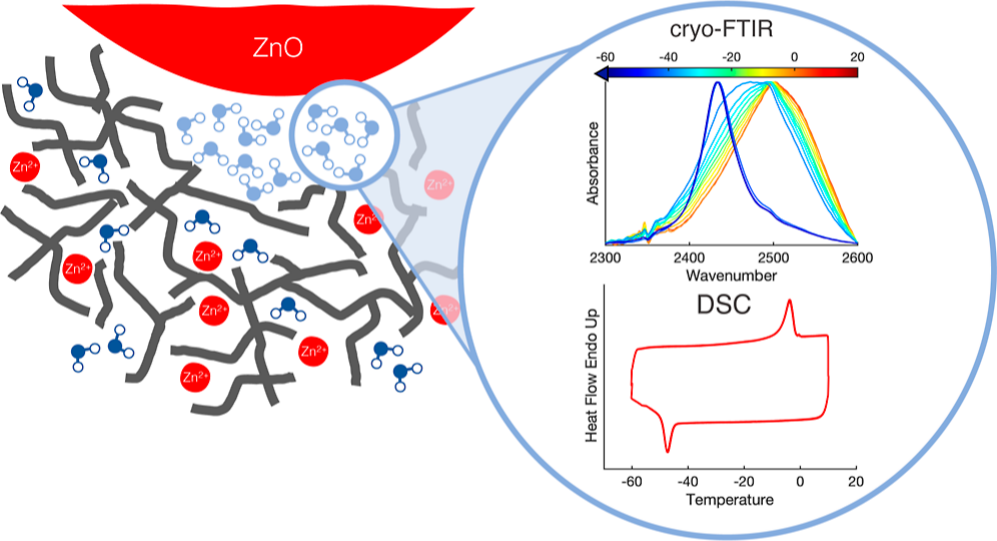

Pigments in oil paint are bound by a complex oil polymer network that is prone to water-related chemical degradation. We use cryo-Fourier-transform infrared spectroscopy and differential scanning calorimetry to study how water distributes inside zinc white oil paint. By measuring water freezing and melting transitions, we show that water-saturated zinc white oil paint contains both liquid-like clustered water and nonclustered water. A comparison of titanium white paint and nonpigmented model systems indicates that water clustering happens near the pigment–polymer interface. The cluster size was estimated in the nanometer range based on the ice melting and freezing temperatures and on the position of the O–D vibration band. As liquid-like water can play a crucial role in the dissolution and transport of ions and molecules, understanding the factors that favor this phenomenon is essential for establishing safe conditions for the conservation of painted works of art.

## Introduction

The behavior and distribution of water in nonhydrophilic environments are important research topics, for instance, in the study of membranes and proteins, nanotubes, organic frameworks, and (super) hydrophobic coatings.^1–5^ Especially in the barrier coating industry, the clustering of water in liquid-like droplets has huge implications for the properties and performance of a coating because the presence of water clusters influences the water diffusion rate, plasticization, and corrosion reactions on metal substrates.^6–11^ Additionally, research on rubber has shown that water inside hydrophobic elastomers such as rubber is distributed as nanoscale droplets.^12–14^ Here, we introduce historical oil paint as a material in which water clustering may have a great influence on material properties and deterioration rates.

Oil paint has been used to create works of art for many centuries.^15^ Fresh oil paint is a suspension of pigment particles in a liquid drying oil such as linseed oil (LO), which itself is a mixture of triglycerides. As a result of oxygen-induced radical auto-oxidation reactions of the unsaturated fatty esters, the oil binder cures and forms a complex, cross-linked polymer network.^16^ Through oxidation and hydrolysis reactions during paint curing and aging, the oil paint polymer network gains polar functional groups such as alcohols, aldehydes, ketones, carboxylic acids, and metal carboxylates, and the polymer network may generate free fatty acids.^17–23^ Depending on factors such as pigment type and paint age, the chemical properties of oil paint may vary wildly. In general, though, oil paint has a rather low water diffusion coefficient (in the order of 10^–13^ m^2^/s) and a low water sorption capacity (ranging between 1 and 10 wt % with a few exceptions).^24–28^

In this work, we investigate the distribution of water inside zinc white (ZnO) oil paint, famous for its use by artists like Mondrian, Picasso, and Pollock in the 19th and 20th centuries and prone to water-related chemical deterioration.^20,29–32^ Zinc white oil paint forms an ionomeric polymer network upon curing, where zinc ions are bound to carboxylate groups on the polymer backbone that form during paint drying.^33^ Of particular concern for the preservation of works of art painted with zinc white is the reaction between free fatty acids and zinc ions that leads to the formation of crystalline zinc soaps.^32^ Recent work indicates that the formation and crystallization kinetics of zinc soaps are strongly influenced by the presence of water.^34–36^ Water may enter the oil paint via humidity in the environment or water-based cleaning and consolidation solutions used in conservation treatments.

We hypothesize that the presence of liquid-like water clusters, as opposed to molecularly distributed water, can open up the possibility of aqueous chemistry inside oil paint layers. For instance, small liquid water domains could play a crucial role in the dissolution and transport of ions and small molecules, processes that stand at the basis of many types of pigment and polymer degradation in oil paint. Therefore, understanding the factors that favor the presence of clustered water and gaining insight into the location of those clusters inside oil paint is hugely relevant for elucidating chemical degradation pathways and the development of safe conservation treatments and storage conditions.

Thus far, only two studies have yielded some information on the distribution of water inside oil paint, both focusing on lead-white oil paint.^26,37^ Using nuclear magnetic resonance spectroscopy and low-frequency dielectric spectroscopy, these reports found indirect evidence that hints at water clustering into liquid domains under high humidity conditions. In our work, the distribution of water was studied by measuring the freezing and melting transitions of water absorbed in zinc white oil paint with differential scanning calorimetry (DSC), commonly used for hydrophilic polymers such as hydrogels to distinguish between bulk and confined water.^38–43^ To obtain more information about the environment of water inside zinc white oil paint, we performed complementary isotope-diluted transmission cryo-Fourier-transform infrared (cryo-FTIR) spectroscopy. Isotope dilution avoids the overlap of symmetric and asymmetric stretch vibrations of water and therefore allows the analysis of the uncoupled O–D stretch vibration in HDO (ν_s_(OD), 2300–2600 cm^–1^), which changes in shape and intensity during the water–ice transition.^44–47^

**Figure 1:**
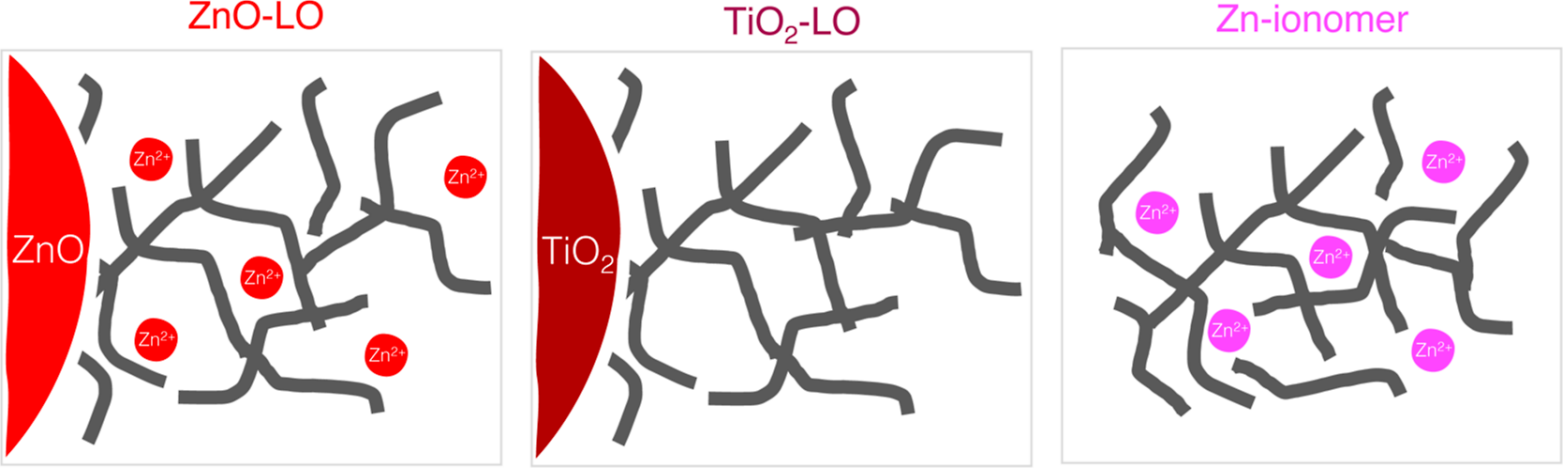
Schematic representations of the three systems compared in this work: zinc white LO paint, titanium white LO paint, and zinc-ionomer system. Gray lines represent the backbone of the polymerized oil network.

Three types of oil paint model systems were investigated: zinc white LO paint (ZnO-LO), titanium white LO paint (TiO_2_-LO), and zinc ionomer (Zn-ionomer). Illustrations of these systems are shown in Figure 1. The comparison of these three paint systems allows for the investigation of the influence of suspended pigment particles and metal ions in the polymer network on the water distribution. Both ZnO-LO and TiO_2_-LO contain pigment particles (zinc oxide and titanium dioxide, respectively), where ZnO-LO forms an ionomeric network and TiO_2_-LO does not. Zn-ionomer simulates the ionomeric binding medium in zinc white oil paint that surrounds the pigment particles. More details on the characterization of the Zn-ionomer model systems can be found elsewhere.^25,33^ In the following sections, the results of the DSC and cryo-FTIR analyses and their consequences for oil paint reactivity will be discussed.

## Methods

### Sample Preparation

Zinc white (ZnO-LO) and titanium white (TiO_2_-LO) oil paints were prepared in a 1:1 w/w pigment/oil ratio. Using a glass muller on a glass slab, 0.102 g of zinc oxide (ZnO) (Aldrich, nanopowder, <100 nm particle size) was mixed in 0.11 mL of LO (Kremer Pigmente, from Sweden, cold-pressed, low acid content) and 0.513 mg of titanium dioxide (TiO_2_) (Huntsman, coated rutile, ±240 nm particle size) in 0.55 mL of LO. This concentration corresponds to pigment volume ratios below the critical pigment volume ratios that are generally reported for zinc white and titanium white oil paints (15 and 20 wt % oil, respectively). Both paints were applied on glass slides using a drawdown bar (30 and 90 μm thickness) and left to cure for 7 days at 60 °C and 12% relative humidity (RH) in the dark. The preparation procedure for the Zn-ionomer model systems was as described by Baij et al. (2018),^25^ see Section A in the Supporting Information. To obtain water-saturated paint samples, small pieces of paint film (1 × 1 cm) were submerged in deionized water for 2–3 days. A set of ZnO-LO (90 μm thickness) paint films was exposed to a 90% RH environment for 5 days.

### Differential Scanning Calorimetry

A PerkinElmer Jade differential scanning calorimeter was used for this study. The temperature program cycled twice between 10 and −60 °C with a rate of 5 °C/min. Paint films (90 μm thickness) were taken out of the water, patted dry with paper tissue before being cut into small pieces, and then placed into sealed aluminum DSC pans. The pans were weighed when empty and after adding the sample to a Sartorius M2P microbalance. To minimize water evaporating from potentially leaking pans, the nitrogen flow in the DSC instrument was turned off during measurements. The aluminum pans were weighed again after the measurement run to check for water evaporation. This data can be found in Section E of the Supporting Information.

### Transmission Cryo-FTIR Spectroscopy

Paint films (30 μm thickness) were left for 2–3 days in a 10–30% D_2_O (Aldrich, 99.9 atoms % D) in a deionized H_2_O solution. The films were taken out of the water, patted dry with paper tissue, and placed between two CaF_2_ windows. The temperature of the sample during the measurements was controlled by using a liquid-nitrogen cryostat (Optistat DN, Oxford Instruments). The sample chamber was filled with 1 bar of helium to ensure rapid and complete thermalization. The temperature of the sample was measured using a PT100 thermocouple. The FTIR spectra were recorded in transmission using a PerkinElmer Spectrum Two FTIR spectrometer with a spectral resolution of 4 cm^–1^. The sample was cooled at a rate of 5 °C/min, and the measurements were collected every 30 (average of 2 spectra) or 90 s (average of 8 spectra). The data processing procedures (normalization and background correction) varied slightly per sample and are indicated in the figure captions.

### Dynamic Vapor Sorption Analysis

Dynamic vapor sorption (DVS) analysis was performed using an automatic multisample moisture sorption analyzer (SPSx- 11m, Projekt Messtechnik). The RH inside the climatic chamber was conditioned by mixing a dry nitrogen gas flow with a gas flow saturated with water. The mass increase of the samples was measured with a 10 min interval on a microbalance (WXS206SDU, Mettler Toledo). The samples (90 μm thickness) were subjected to an initial drying step at 0% RH for 300 h before the start of the sorption experiment. The RH was varied in 10% steps every 50 h up to 90%, with a final step between 90 and 95% at 22 °C. Equilibrium sorption was assumed to have been reached when there was no mass change of >0.001% over a period of 60 min or a maximum step time of 50 h.

## Results and Discussion

### DSC Measurements on Water-Saturated Paint Films

**Figure 2:**
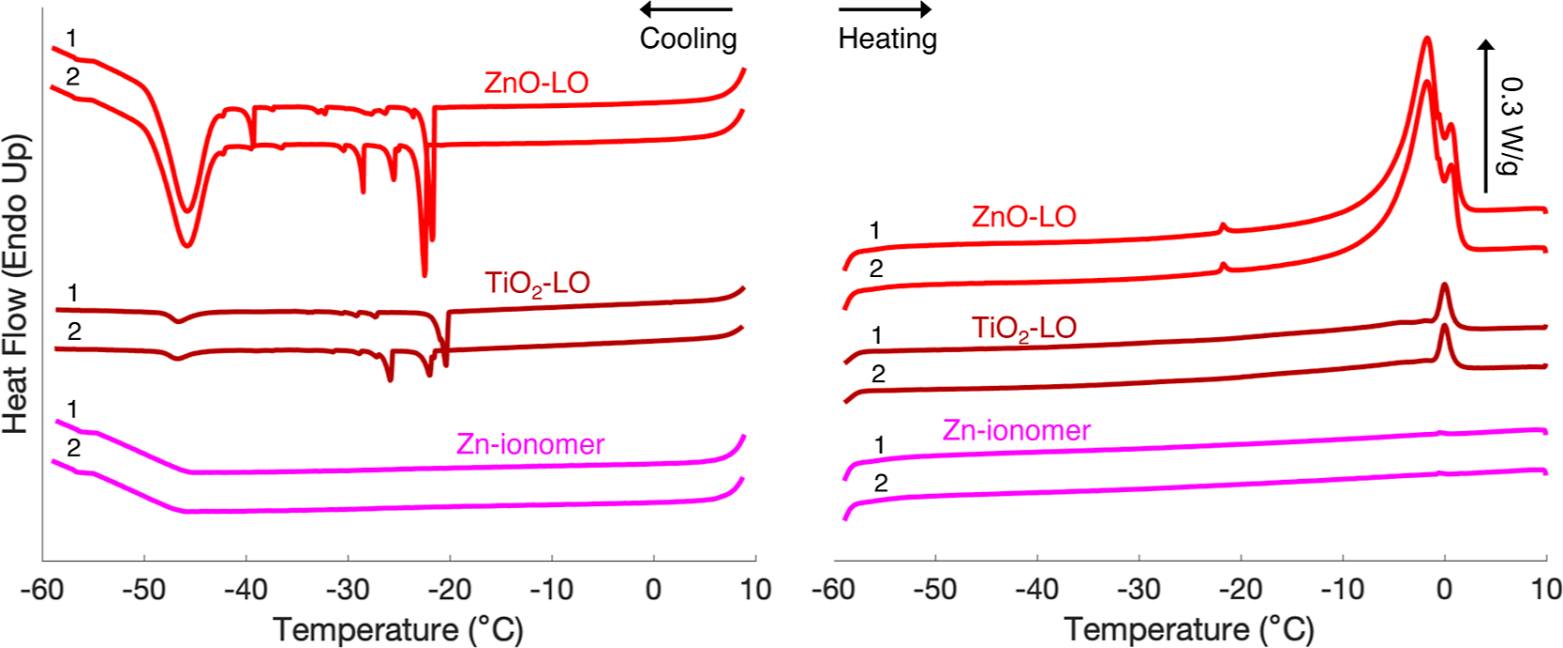
Mass-corrected DSC thermograms of water-saturated ZnO-LO, TiO_2_-LO, and Zn-ionomer films. Two heating and cooling cycles between 10 and −60 °C were performed with a scan rate of 5 °C/min. Numbers 1 and 2 refer to the first and second DSC runs. Full DSC thermograms are reported in Section D of the Supporting Information.

Films of cured oil paint were placed in water until they reached saturation. Sorption isotherms of the oil paint films can be found in Figure S1 in the Supporting Information. Freezing and melting transition data are reported in Section C in the Supporting Information in the Supporting Information. The thermogram of ZnO-LO (Figure 2) showed two types of freezing transitions: a broad freezing peak with an onset at approximately −43 °C and several sharp peaks in the region of −20 to −35 °C. This latter region is similar to where supercooled bulk water freezes when measured at the same scan rate (Figure S9). The sharp peaks are interpreted as free water on the surface of the paint films.^14,48^ Despite efforts to dry the surface of the films prior to measurements, these peaks were difficult to avoid when measuring thin films and were featured in the thermograms in an irreproducible manner. Free water undergoes a melting transition at 0 °C, which corresponds to the second of the two melting peaks for ZnO-LO. The very low freezing transition at −43 °C is interpreted as the crystallization of confined water.^49,50^ The melting transition of this confined water corresponds to the first melting peak in the thermograms of ZnO-LO, with an onset well below 0 °C. The freezing peak at approximately −43 °C in ZnO-LO paint films was highly reproducible (Table S2). The exact position, however, varied by several degrees depending on the scan rate (Table S3). Despite a lower water content, the same freezing/melting behavior was observed in the thermogram of TiO_2_-LO: a freezing peak at −44 °C, irregular freezing peaks between −20 and −30 °C, and split melting peaks below and at 0 °C. The nonpigmented Zn-ionomer did not exhibit any freezing/melting transitions, while it did absorb water at a similar concentration as ZnO-LO at 95% RH (see Figure S1).

### Cryo-FTIR Spectroscopy on Water-Saturated Paint Films

**Figure 3:**
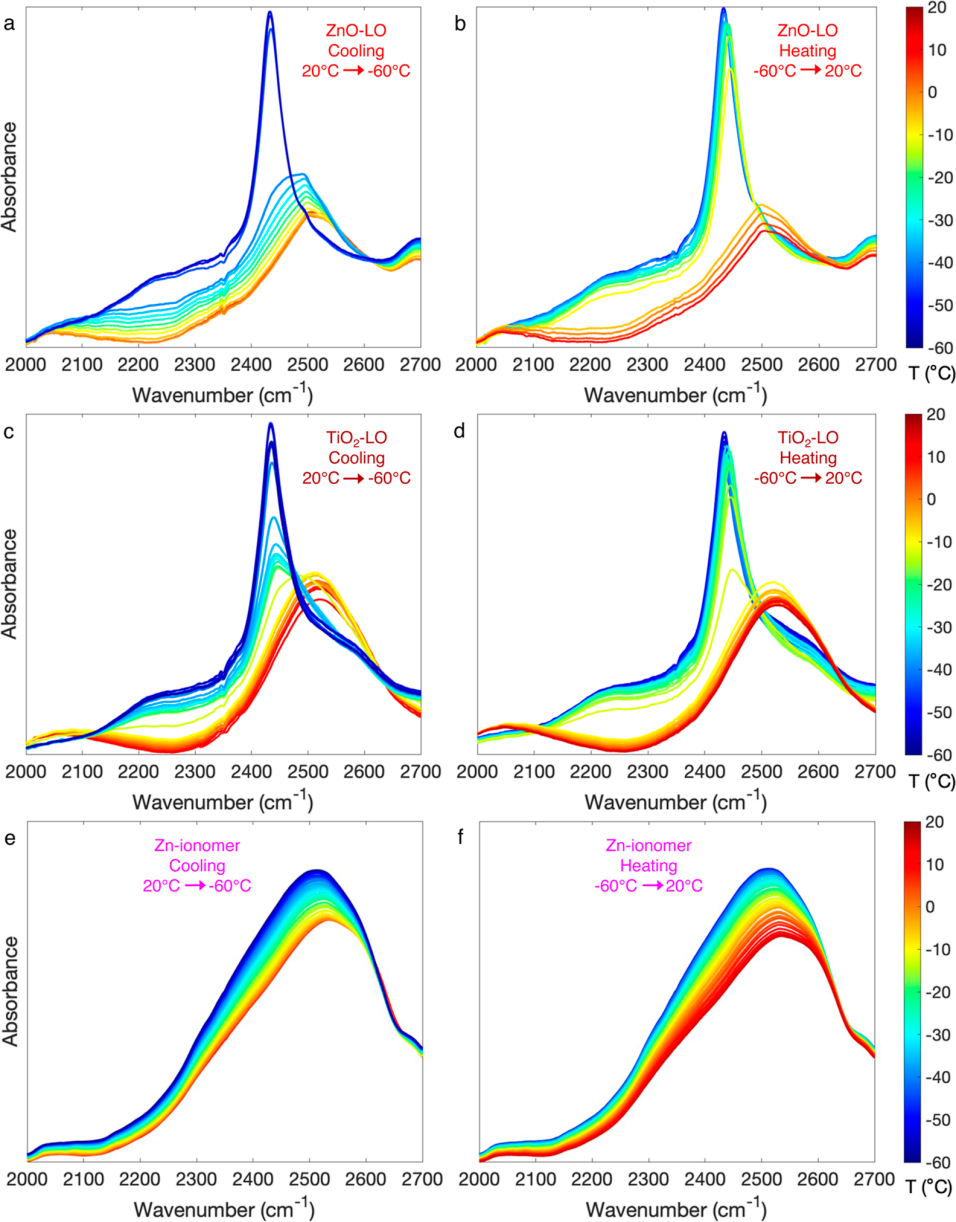
O–D stretch vibration region of the transmission FTIR spectrum of water-saturated ZnO-LO (a,b), TiO_2_-LO (c,d) (10% D_2_O in H_2_O), and Zn-ionomer (e,f) (30% D_2_O in H_2_O) during cooling and heating. Curve colors correspond to the temperature of the sample. Linear baseline subtraction was performed between 1900 and 4000 cm^–1^, and spectra were normalized on ν_s_ (CH)CH_2_ at 2854 cm^–1^.

**Figure 4:**
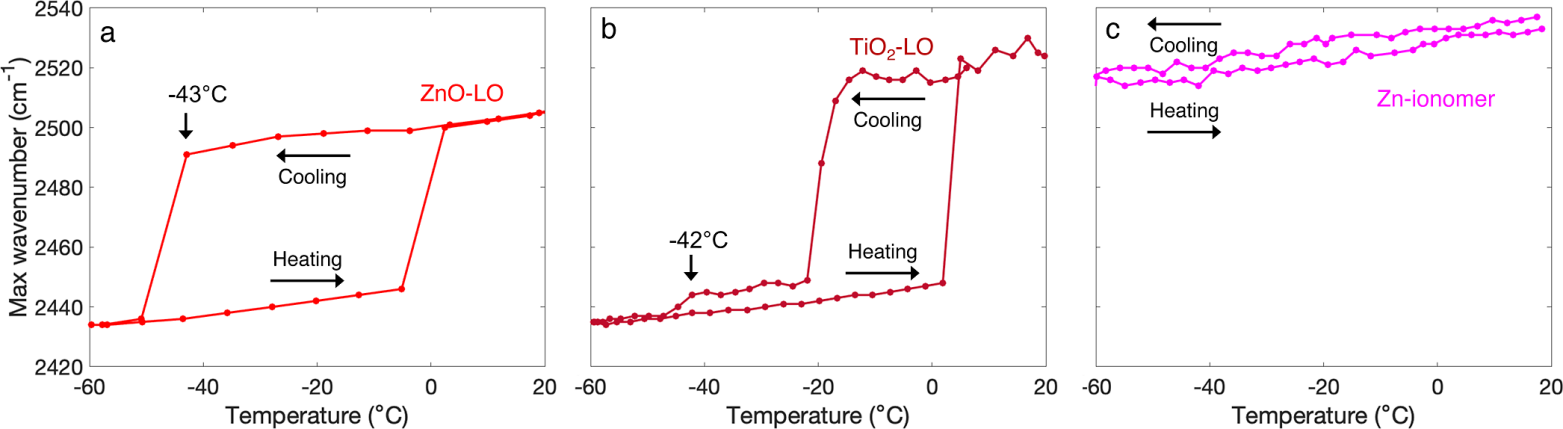
O–D vibration band maximum profiles of water-saturated ZnO-LO (red), TiO_2_-LO (dark red), and Zn-ionomer (magenta). The scanning rate was 5 °C/min.

The phase transitions that were observed with DSC could be confirmed with cryo-FTIR spectroscopy on thin films of the paint systems (approximately 30 μm thick) soaked in 10–30% D_2_O in H_2_O. Moreover, the appearance of sharp OD bands at 2430 cm^–1^ (Figure 3) confirmed that the detected transitions are indeed corresponding to ice formation and melting.^47^ The position of the ν_s_(OD) band maximum as a function of the temperature is shown in Figure 4. In ZnO-LO, there was a water freezing transition at −43 °C and a melting transition at −5 °C (Figure 4a). The TiO_2_-LO profile in Figure 4b indicates a two-step transition, with the first step at −12 °C and the second at −42 °C. These transitions correspond to the freezing of two types of water, as seen in the DSC thermograms: free water at −12 °C and confined water at −42 °C. ν_s_(OD) shifted back to high wavenumbers at 2 °C. Due to limitations in temperature resolution, separate melting transitions for confined and bulk water could not be resolved with cryo-FTIR spectroscopy. The Zn-ionomer profile in Figure 4c does not exhibit any phase transitions.

There are interesting differences in the shape and position of the OD stretch vibration band between the three paint systems. It is important to note that the OD band contains a contribution of deuterated alcohol groups that are part of the oil polymer network. The alcohol content can be estimated by comparing the OH stretch vibration band area in dried films (assumed to correspond to alcohol OH stretch vibrations only) and in H_2_O-saturated films. Using this approach, the alcohol contribution was estimated to form 18, 56, and 20% of the total OD absorbance for ZnO-LO, TiO_2_-LO, and Zn-ionomer, respectively (see Figure S11). For this reason, it is not possible to analyze the OD band shape in great detail, but some additional observations can still be made. In both ZnO-LO and TiO_2_-LO, a second band at 2230 cm^–1^ appeared during cooling, which is assigned to the second overtone of a librational mode (3ν_L_) of H_2_O ice (*I*_h_).^51,52^ Furthermore, Figure 4 clearly shows that all three profiles exhibit a red shifting trend in ν_s_(OD) with decreasing temperature.^45^

### Environment of Freezable Water in Oil Paint

We observed that pigmented ZnO-LO and TiO_2_-LO paints contain freezable water that underwent a freezing transition around −45 °C, whereas the nonpigmented system that models the binding medium of zinc white paint, Zn-ionomer, only contains water that does not freeze above −60 °C. These observations indicate that there are two types of water inside zinc white oil paint: nonfreezable water in the binding medium and freezable clustered water located near the pigment–oil interface. The hypothesized void where this clustered water could reside between the pigment particle surface and the oil polymer network can be compared to the concept of interphase in the field of nanocomposite materials.^53^ Furthermore, water accumulation at the surface of pigment particles in coatings is discussed regularly in the literature as it negatively impacts the low water permeability properties of barrier coatings.^7,11^

The remarkably low freezing point of water in our systems provides interesting additional clues about the nature and environment of water clusters in oil paint. First, the strong degree of supercooling is proof that the clustering water is forming discrete droplets as opposed to a connected network of water channels. Only in small discrete volumes, the probability of ice nucleation will be low enough to reach this degree of supercooling.^14^ Furthermore, recent work by Hakimian et al. showed that soft confinement is a critical factor for supercooling of water to such low temperatures.^54^ They were able to cool down water in octane oil to −42 °C in pores with a 2–4 nm diameter, whereas water directly in contact with the anodized aluminum oxide membrane pore wall did not supercool below −8 °C. These observations suggest that the confined water in pigmented oil paint is not just adsorbed in pores or cracks inside pigment particles but is still in at least partial contact with the oil polymer network.

A curious observation in our systems is the large hysteresis between the freezing and melting points of water. While some degree of hysteresis due to the kinetics of nucleation is expected, the hysteresis observed here for ZnO-LO is much larger compared to the hysteresis generally reported for water confined in mesoporous silica gel.^55^ A very interesting comparison to our systems is provided by studies of butyl rubber. A similarly large hysteresis between the freezing temperature (*T*_f_ = −38 °C) and melting temperature (*T*_m_ = −6.5 °C) of water inside butyl rubber was found, similar to our paint systems (*T*_f_ = −43 °C and *T*_m_ = −5 °C for ZnO-LO).^14^ According to Neffati et al., the large hysteresis is due to the swelling of the elastic matrix of the rubber.^12,14^ LO paint films are also known to experience swelling in water (5%), although this swelling is relatively minor compared to other solvents.^56^ Moreover, water inside rubber was found to disperse as droplets around hydrophilic sites.^12,14,48^ In fact, the investigated rubbers in the studies by Neffati et al. contained ZnO particles (5 wt %), which were suggested to act as hydrophilic sites where the water droplets form.^14,48^

### Water Cluster Size Estimate

It is insightful to estimate the size of water clusters in oil paint by comparison to similar confined-water systems in the literature. A first estimate of the water cluster size can be made based on the confinement-induced melting point depression of ZnO-LO. The melting point is determined only by thermodynamics, as opposed to the freezing point which is also influenced by the kinetics of nucleation.^57^ Melting point depression can also be affected by dissolved ions or small organic molecules. Melting point depressions of several tens of Kelvins are reported for high salt concentrations (multiple M),^58^ and lower concentrations of NaCl (up to 0.2 M) in mesoporous confinement may lead to melting point depressions of 20% on top of confinement-related depression.^59^ If we assume that confinement is the major factor affecting the melting point of water in our systems, it is possible to calculate the radius of a spherical crystal that is surrounded by its own liquid during melting following the Gibbs–Thomson equation:
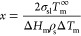
1

Here, *x* is the radius of a spherical crystal, σ_sl_ corresponds to the ice–water interfacial energy, *T*_m_^∞^ to the melting temperature of bulk water, Δ*H*_m_ to the latent heat of fusion of ice, ρ_s_ to the density of ice, and Δ*T*_m_ to the melting point depression.^60^ The latent heat of fusion decreases with decreasing melting temperatures.^61^ As the melting temperature here is only shifted by 5 K, it is acceptable to use the latent heat of fusion at 273 K. These assumptions lead to a water cluster diameter 2*x* of approximately 16 nm (σ_sl_ = 22.8 × 10^–3^ J m^2^, Δ*H*_m_ρ_s_ = 3.06 × 10^8^ J/m^3^, and Δ*T*_m_ = 5 K).

Estimating the size of water clusters is challenging in a complex environment, such as oil paint. Therefore, in order to provide a reasonable range of cluster sizes, we discuss two methods here. In addition to the thermodynamic approach stated above, we discuss a kinetic approach as well. Focusing on the freezing transition, several studies have discussed the relationship between freezing point depression and water cluster size. Neffati et al. based their size estimate of water clusters inside butyl rubber on the thermoporosimetry method by Brun.^14,62,63^ They found that a freezing point of −38 °C corresponds to a cluster diameter of 3.4 nm, which they confirmed using ^2^H NMR relaxometry.^14^ Using the same method, the water cluster diameter in ZnO-LO would be approximately 3 nm. Pelster et al.^48,64^ reported a freezing temperature of water in butyl rubber of −43 °C and an experimentally determined cluster diameter of 2.6 nm based on thermally stimulated depolarization current and small-angle X-ray scattering analysis, while Spehr et al. reported a supercooling of 50 °C for reverse micelles with a diameter just below 2 nm.^64^ Using a different approach, Dokter et al. showed that the IR spectral features of ice in reverse micelles are dependent on the number of water molecules inside the micelle. Water in reverse micelles containing fewer than 200 molecules forms amorphous ice with a broad spectral feature at around 2500 cm^–1^, whereas groups of over 200 water molecules form crystalline ice with a sharp band at 2435 cm^–1^. Based on the presence of the sharp O–D band at 2435 cm^–1^ in the cryo-FTIR spectrum of ZnO-LO, we can infer a minimum cluster size of 200 water molecules,^46^ which corresponds to a spherical volume with a diameter of 2.24 nm based on the molar volume of water. This conclusion is in line with the observation that water crystallization inside mesoporous silica gel with a pore diameter of 2.1 nm was inhibited.^65^

Combining all the evidence, based on the melting and freezing points of water in ZnO-LO, we estimate the diameter of the water clusters in this oil paint to be between 2 and 16 nm. If the confined water clusters in ZnO-LO are indeed of nanometer size, we should expect to see evidence of this confinement in the FTIR spectra. The ν_s_(OD) band is known to shift to a higher frequency with the decreasing size of confined water clusters.^66,67^ A blueshift for reverse micelles is reported from 2512 cm^–1^ for bulk water to 2565 cm^–1^ for interfacial water, which is water that interacts directly with the micelle boundary.^66^ ν_s_(OD) at room temperature for ZnO-LO is located at 2505 cm^–1^, very similar to that of bulk water.^47^ This band position suggests that a large fraction of the water in ZnO-LO is bulk-like liquid water and that the size is likely to be at the high end of our 2–16 nm estimate. Conversely, in the case of the Zn-ionomer, it seems likely that the cluster size is below the 200-molecule limit as both DSC and cryo-FTIR analysis indicate that the water present inside the Zn-ionomer does not undergo a freezing transition above −60 °C. Data reported by Spehr et al. suggests that a freezing point below −60 °C would correspond to a very small cluster radius of only a few Å.^64^ A smaller water cluster size in Zn-ionomer compared to ZnO-LO is also supported by the strong blueshift of ν_s_(OD) for Zn-ionomer (2534 cm^–1^) compared to ZnO-LO (2502 cm^–1^). This blueshift in Zn-ionomer indicates that Zn-ionomer contains a larger contribution of interfacial water. For these reasons, we classify the water in Zn-ionomer as nonfreezable and molecularly distributed.

### Implications of Water Clusters on Chemical Reactivity and Painting Conservation

Our experiments indicate that water in zinc white oil paint is heterogeneously distributed as molecularly dispersed water in the oil polymer network and liquid-like water near the pigment–polymer interface. In addition, we see evidence that the presence of zinc ions in the polymer network positively influences the water sorption capacity of the polymer, given the low water sorption in titanium white paint. This conclusion is supported by studies on polyethylene Zn-ionomer systems, where the presence of zinc ions increased the water sorption capacity.^68,69^ These findings have large implications for our understanding of chemical changes in oil paint pigmented with zinc white or other pigments. We imagine that liquid-like water located near the ZnO surface can stabilize and/or dissolve ions and small polar molecules. As such, water clusters could accelerate reactions between pigments and carboxylic acid groups or other functionalities that break down the pigment or facilitate the recrystallization of pigments to new mineral phases.^20,70–77^ In addition, ionic species could diffuse through regions of the paint in solvated form when liquid water is present, while this diffusion is likely to be much slower in the absence of liquid water.

A crucial step toward understanding the practical implications of these findings in a conservation context is to know which environmental conditions lead to water cluster formation in oil paint. The measurements in this work were performed on water-saturated paint films. It is important to know whether clustered water may form in oil paints in a typical museum climate, where humidity from the air is the primary source of moisture. For that goal, we performed DSC measurements on ZnO-LO conditioned at 90% RH (Figure S10). No water freezing–melting transitions were detected under those conditions. This result suggests that water clusters arise only in (near)-saturated conditions. These conditions could arise in the surface region of paint during liquid water exposure, for instance, during cleaning and consolidation interventions using aqueous solutions. Generally, conservators take the utmost care to control water exposure during these types of interventions, for example, by using gels or tissues to deliver a solution to a painted surface. In light of the findings presented here, our future research will focus on investigating the rate and location of clustered water formation in oil paint during treatment and on the behavior of paints with different pigmentation. In addition, further quantification of the ratio of freezable to nonfreezable water would provide additional insights into the behavior of moisture in oil paint. In order to calculate the ratio between the two states of water, a reliable measure of the moisture content at saturation is necessary. We attempted to measure the moisture content of the paint films by gravimetry but did not achieve reproducible results. Gravimetry is challenging because of the low moisture content of oil paint, evaporation, and the presence of free water on the surface. Future research will focus on addressing this issue. Lastly, in an effort to provide conservators with tools to guide decision-making around treating sensitive oil paint, we are exploring noninvasive techniques for monitoring water-based conservation treatments.

## Conclusions

In this work, a complementary combination of DSC and cryo-FTIR spectroscopy was successfully used to detect heterogeneity in water distribution in oil paint at the nanoscale. We have shown that zinc white oil paint contains molecularly dispersed water in the polymerized oil binder and liquid-like water clusters with nanometer size near the pigment–polymer interface under saturated conditions. The presence of liquid-like water clusters has important implications for our understanding of chemical processes in oil paint. These findings change our perspective on oil paint chemistry as they prompt us to consider aqueous chemistry when studying paint degradation. Furthermore, the results lead to a wide range of new questions, both fundamental and practical. Future research will focus on extending the research to oil paints with different pigments and degrees of aging. Moreover, noninvasive monitoring techniques may be used to establish object-specific guidelines. Finally, investigations into the effect of clustered water on pigment–binder reactions will help establish the important link between water exposure and reactivity in paintings, which is critical information for establishing safe parameters for storage, display, and treatment of painted works of art.

## Supporting Information Available

Additional experimental details, water sorption isotherms, melting and freezing points determined by DSC, DSC thermograms, weight loss during DSC, and transmission FTIR spectra at room temperature (PDF)
