# Supplementary material for: Nanoconfined Water Clusters in Zinc White Oil Paint
Source: J Phys Chem C Nanomater Interfaces. 2023 Sep 15;127(38):19269–77. doi: 10.1021/acs.jpcc.3c04720 (PMC10544026; doi:10.1021/acs.jpcc.3c04720)
Supplement: Supplementary file 1 — jp3c04720_si_001.pdf [file jp3c04720_si_001.pdf]

Supporting Information

Nanoconfined Water Clusters in Zinc White Oil Paint

Jorien R. Duivenvoorden[1,2]*, Federico Caporaletti[3], Sander Woutersen[1], Katrien Keune[1,2] and Joen J. Hermans[1,2,4]

[1] Van 't Hoff Institute for Molecular Sciences, University of Amsterdam, Amsterdam

[2] Conservation & Science, Rijksmuseum, Amsterdam

[3] Laboratory of Polymer and Soft Matter Dynamics, Experimental Soft Matter and Thermal Physics, Université Libre de Bruxelles, Brussels

[4] Conservation & Restoration, Amsterdam School of Heritage, Memory and Material Culture, University of Amsterdam, Amsterdam

*Corresponding author: j.r.duivenvoorden@uva.nl

Contents

A. Additional experimental details .... p. S1
B. Water sorption isotherms .... p. S2
C. Melting and freezing points determined by DSC .... p. S2
D. DSC thermograms .... p. S4
E. Weight loss during DSC .... p. S8
F. Transmission FTIR at room temperature .... p. S9

A. Additional experimental details

Zinc sorbate preparation

Zinc sorbate (ZnSo) was synthesised by adding 0.559 g sorbic acid (Sigma, ≥99.0%) and 1 mL triethylamine (Sigma-Aldrich, ≥99.5%) to 20 mL deionised water at 60°C. 1.016 g zinc nitrate hexahydrate (Sigma-Aldrich, ≥99.0%) was dissolved in 5 mL deionised water. This solution was added dropwise to the sorbic acid solution and left stirring for 20 minutes. The precipitated ZnSo was separated by vacuum filtration, washed with water and acetone before drying overnight over phosphorus pentoxide under vacuum. No unexpected or unusually high safety hazards were encountered. The zinc ionomer model systems (Zn-ionomer) were prepared by mixing 0.074 g ZnSo in 0.53 mL LO with mortar and pestle. This mixture was applied on glass slides using a drawdown bar (30 and 90 μm) and left to cure overnight in the dark at 150°C.

Transmission Fourier-transform infrared (FTIR) spectroscopy at room temperature

Supporting measurements were performed using transmission FTIR spectroscopy at room on a Frontier spectrometer (Perkin Elmer). Individual spectra were collected as a single scan and at 4 cm^{-1} resolution. The paint films (30 μm thickness) were placed directly in the infrared beam path without calcium fluoride windows by taping the films over the hole in a metal plate. The data processing details (normalisation and background correction) are indicated in the figure caption.

B. Water sorption isotherms

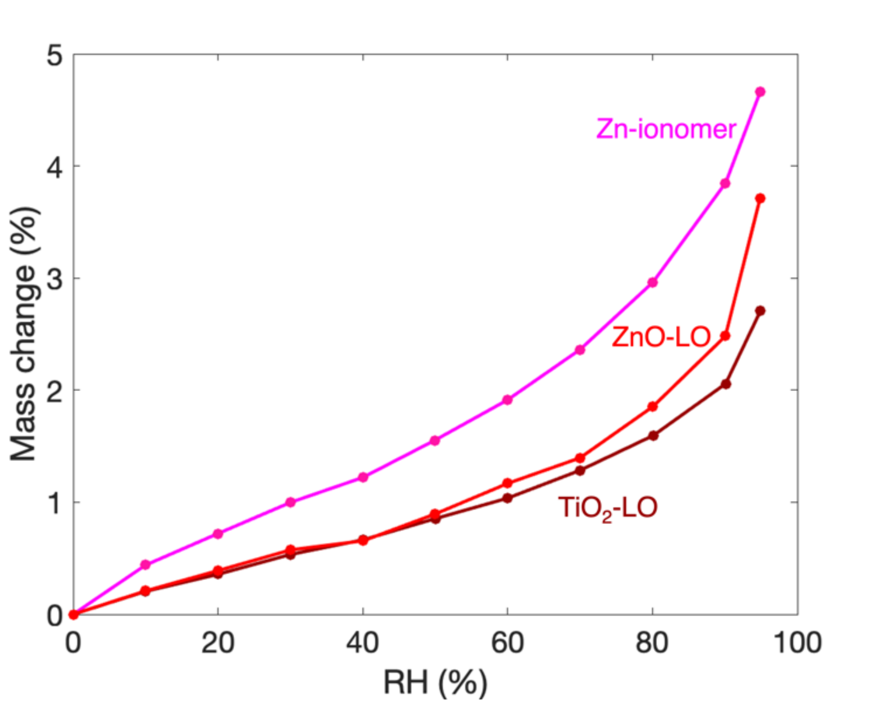

Figure S1. Water sorption isotherms showing the mass change (%) of ZnO-LO, TiO_2-LO and Zn-ionomer films as a result of exposure to humid air between 0 and 95% RH.

C. Melting and freezing points determined by DSC

Table S1. Melting and freezing points of ZnO-LO, TiO_2-LO and Zn-ionomer.

Sample name	ZnO-LO	TiO_2-LO	Zn-ionomer
Onset cold freezing peak (°C)			
Cycle 1	-43.1	-44.4	-
Cycle 2	-43.2	-44.3	-
Minimum cold freezing peak (°C)	-45.8	-46.6	-
Cycle 1	-45.8	-46.6	-
Cycle 2			
Onset melting peak 1 (°C)			
Cycle 1	-5.2	-6.5	-
Cycle 2	-5.2	-6.8	-
Maximum melting peak 1 (°C)			
Cycle 1	-1.7	-4.1	-
Cycle 2	-1.7	-3.8	-
Onset melting peak 2 (°C)			
Cycle 1	-2.2	-0.8	-
Cycle 2	-2.4	-0.7	-
Maximum melting peak 2 (°C)			
Cycle 1	-0.7	0.0	-
Cycle 2	-0.7	0.0	-

Table S2. Melting and freezing points of two more ZnO-LO paint films from the same batch.

Sample name	ZnO-LO-2	ZnO-LO-3
Onset cold freezing peak (°C)		
Cycle 1	-42.9	-42.6
Cycle 2	-42.9	-42.9
Minimum cold freezing peak		
(°C)	-45.9	-45.0
Cycle 1	-45.6	-45.7
Cycle 2		
Onset melting peak 1 (°C)		
Cycle 1	-6.0	-5.2
Cycle 2	-5.6	-5.0
Maximum melting peak 1 (°C)		
Cycle 1	-1.8	-1.2
Cycle 2	-1.8	-1.3
Onset melting peak 2 (°C)		
Cycle 1	-1.1	-2.3
Cycle 2	-1.2	-3.1
Maximum melting peak 2 (°C)		
Cycle 1	-0.3	-0.2
Cycle 2	-0.4	-0.3

Table S3. Melting and freezing points of ZnO-LO with different DSC scanning rates.

Scanning rate	5 °C/min	10 °C/min	20 °C/min
Onset cold freezing peak			
(°C)	-43.2	-42.6	-45.0
Cycle 1	-43.2	-42.6	-45.1
Cycle 2			
Minimum cold freezing peak			
(°C)	-45.3	-44.5	-46.9
Cycle 1	-45.4	-45.5	-47.0
Cycle 2			
Onset melting peak (°C)			
Cycle 1	-4.4	-4.2	-3.3
Cycle 2	-4.4	-4.2	-3.4
Maximum melting peak (°C)			
Cycle 1	-1.5	-0.9	-0.3
Cycle 2	-1.5	-0.9	-0.3

D. DSC thermograms

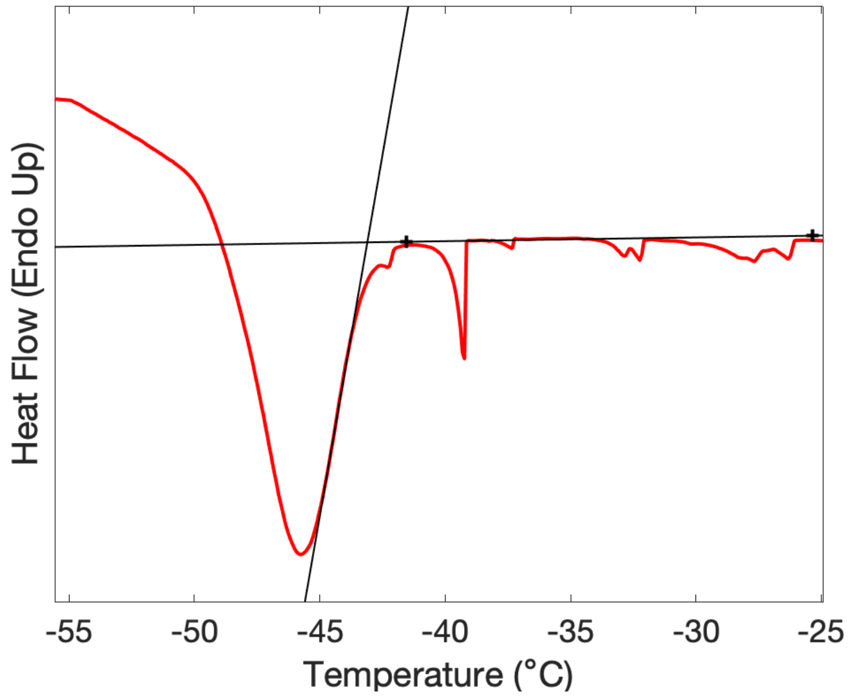

Figure S2. Example of calculation of onset of a peak. The onset is defined as the point where the tangent intersects the baseline.

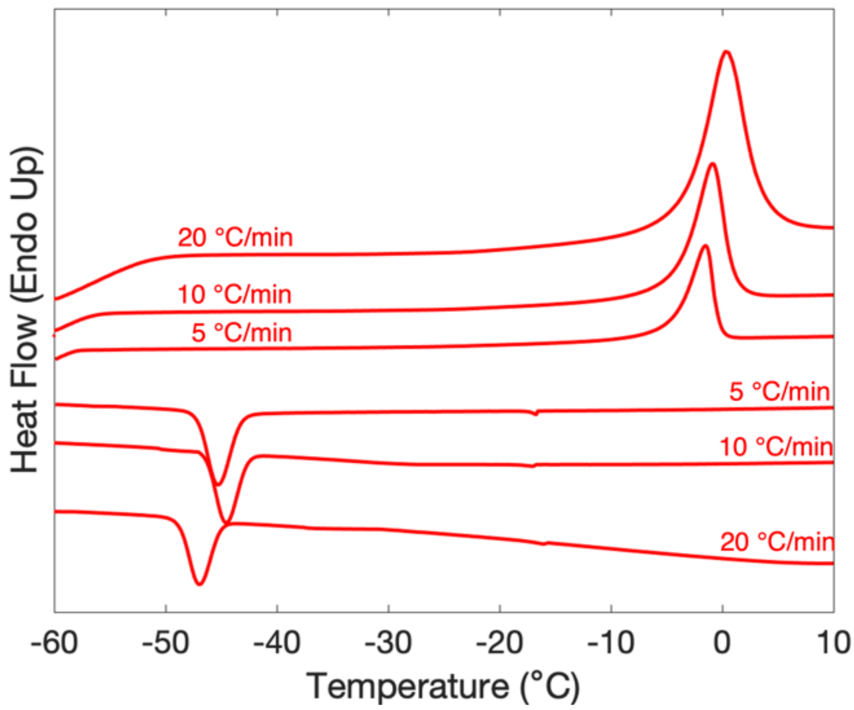

Figure S3. Freezing and melting transitions of ZnO-LO with different scanning rates.

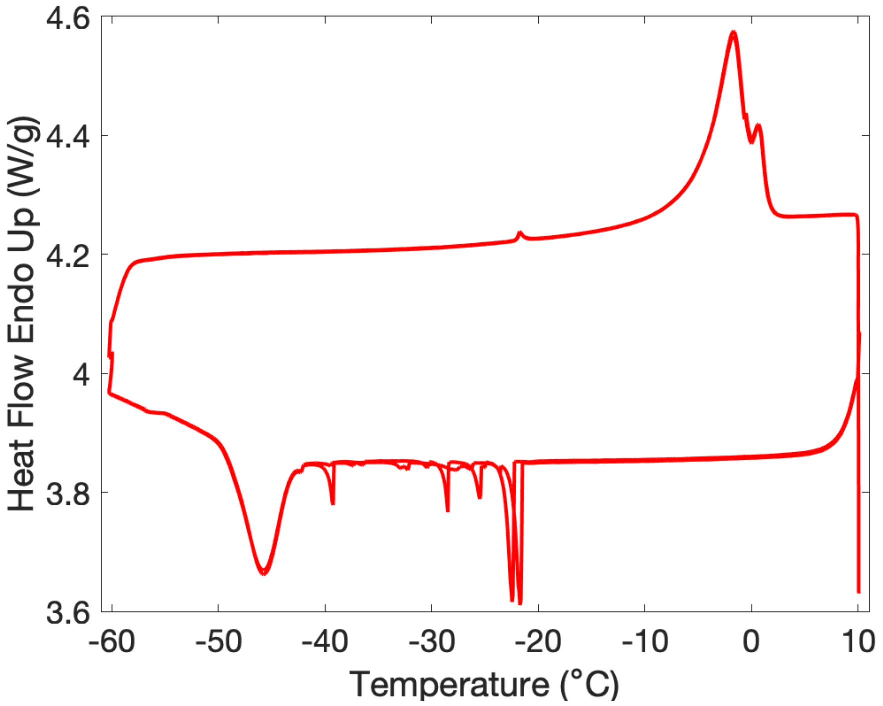

Figure S4. DSC thermogram of ZnO-LO corrected for the sample weight (5.67 mg).

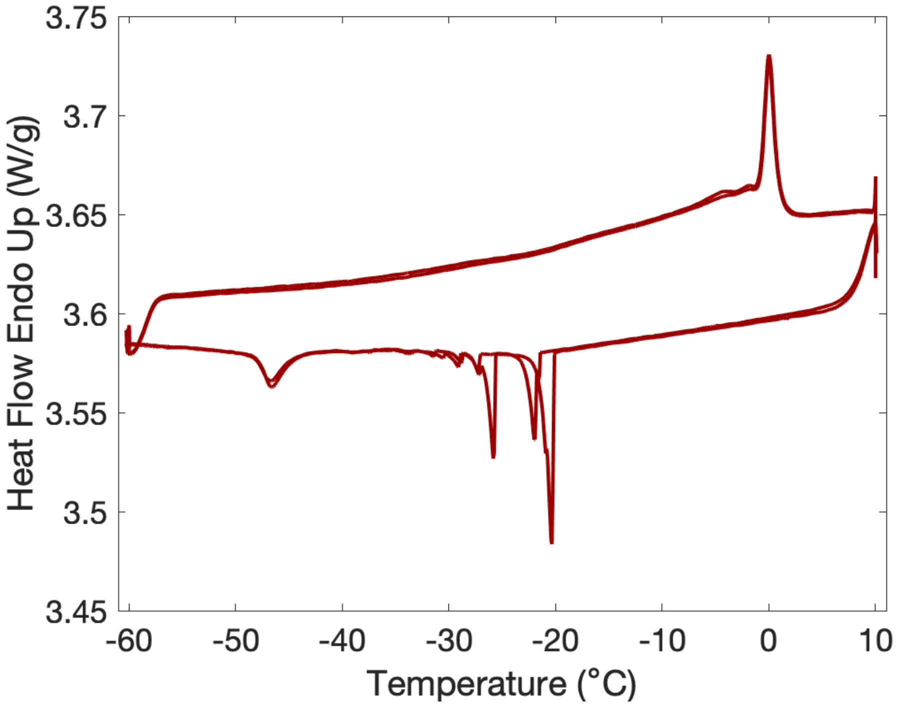

Figure S5. DSC thermogram of TiO_2-LO corrected for the sample weight (5.83 mg).

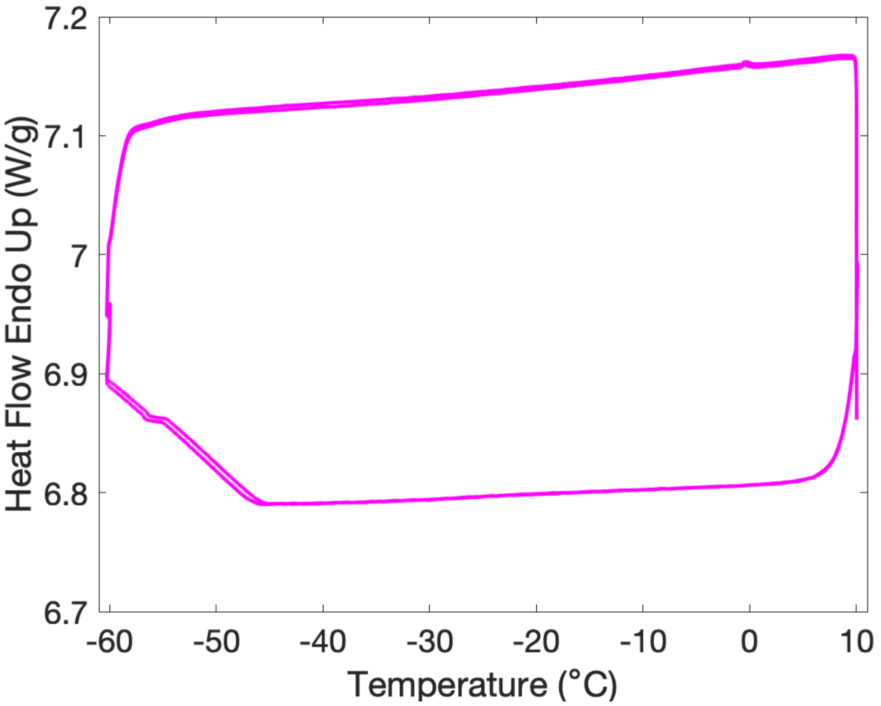

Figure S6. DSC thermogram of Zn-ionomer corrected for the sample weight (4.71 mg).

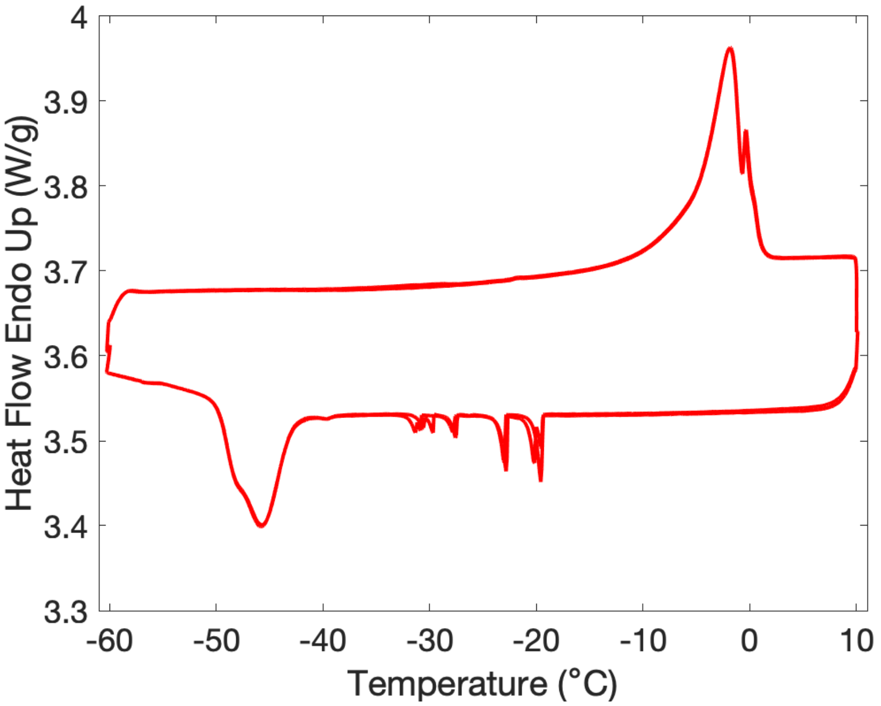

Figure S7. DSC thermogram of ZnO-LO-2 corrected for the sample weight (5.51 mg).

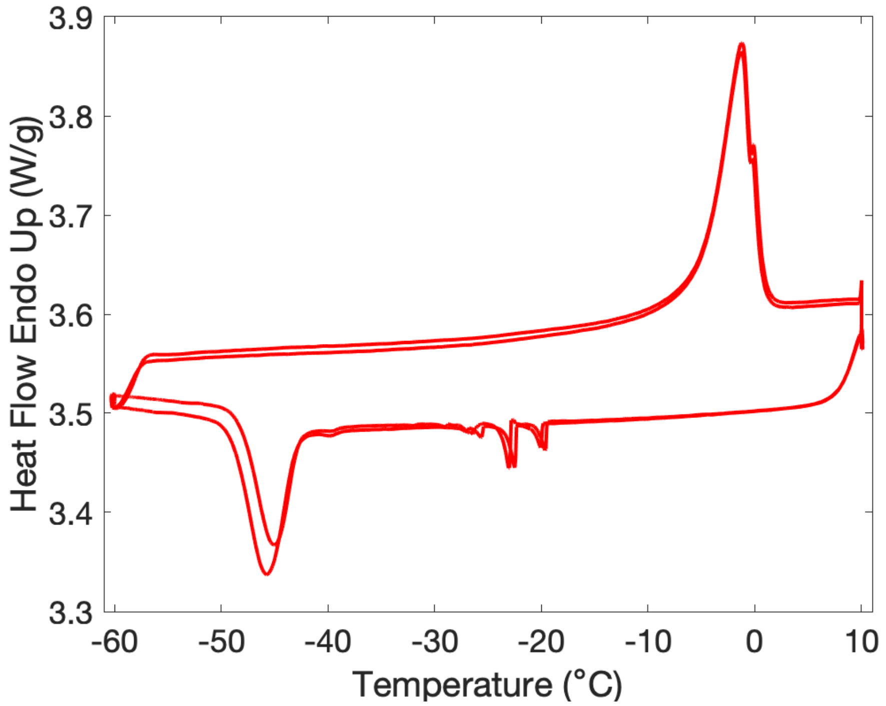

Figure S8. DSC thermogram of ZnO-LO-3 corrected for the sample weight (5.61 mg).

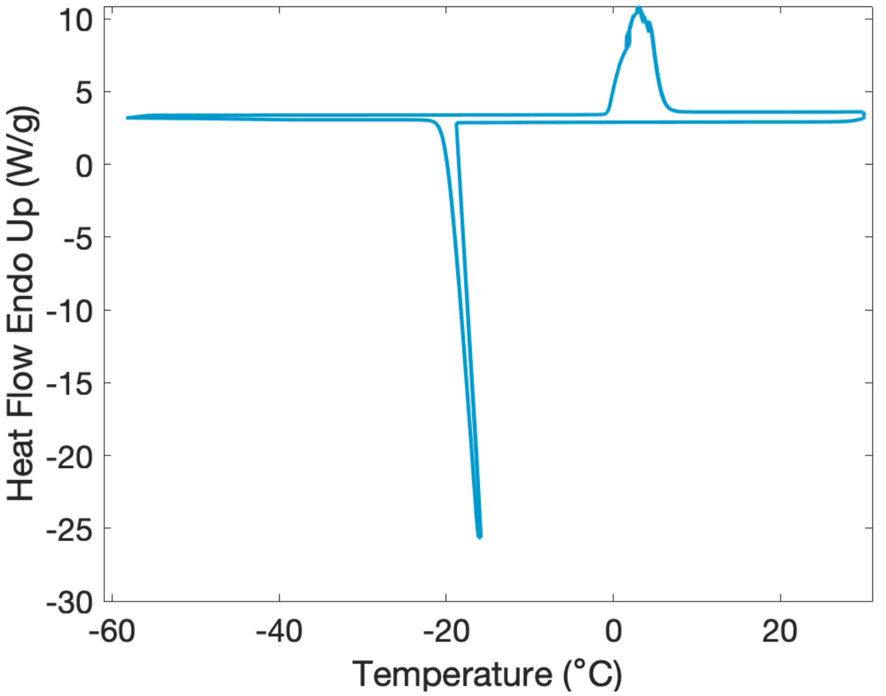

Figure S9. DSC thermogram of bulk demineralised water corrected for the sample weight (5.5 mg).

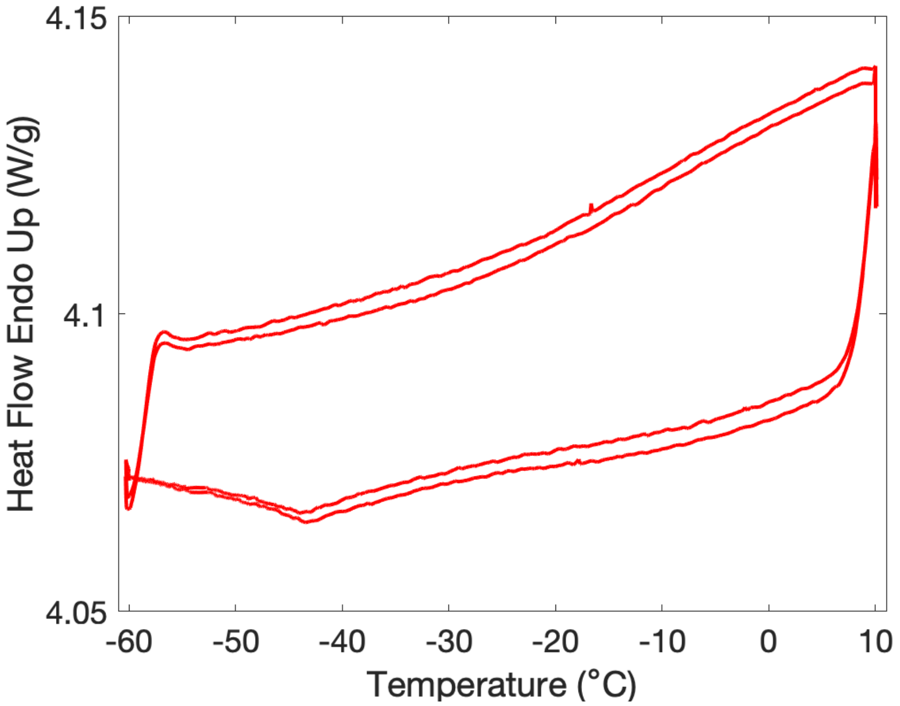

Figure S10. DSC thermogram of ZnO-LO conditioned at 90% RH corrected for the sample weight (4.94 mg).

E. Weight loss during DSC

Table S4. Weight loss during DSC measurements.

Sample name	ZnO-LO	TiO_2-LO	Zn-ionomer	ZnO-LO-2	ZnO-LO-3
Sample weight (mg)	5.67	5.83	4.71	5.51	5.61
Weight sample + DSC pan before measurement (mg)	55.50	55.55	54.88	54.88	55.17
Weight sample + DSC pan after measurement (mg)	55.42	55.53	54.87	54.87	55.14
Weight loss (%)	0.15	0.03	0.01	0.02	0.06

F. Transmission FTIR at room temperature

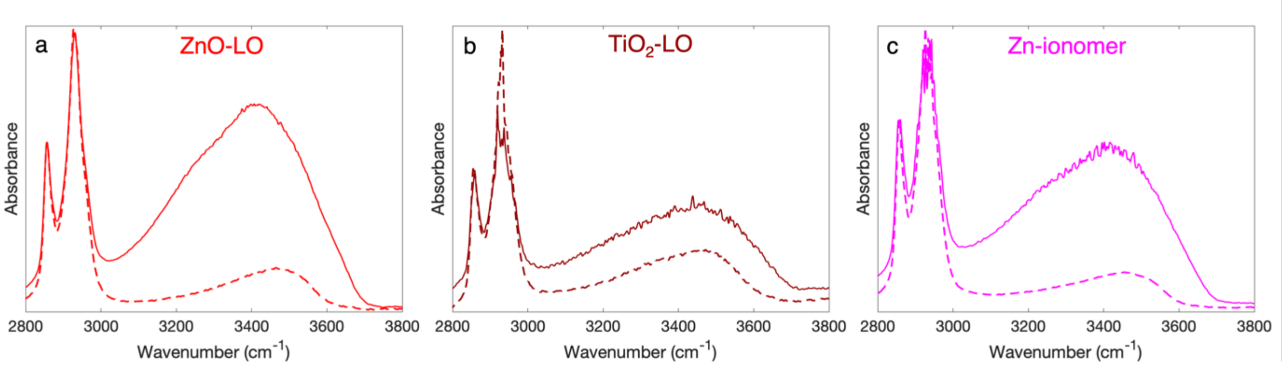

Figure S11. Transmission FTIR spectra showing OH stretch band of saturated paint film (continuous line) and dried paint film (dashed line) (1 minute at 130°C) for ZnO-LO (a), TiO_2-LO (b) and Zn-ionomer (c). Normalization on ν_s (CH)CH_2 at 2859 cm^{-1}. The alcohol contribution is estimated from the ratio of the integrated OH stretch band of the dried film compared to the saturated film. The estimated alcohol contribution corresponds to 18%, 56% and 20% for ZnO-LO, TiO_2-LO and Zn-ionomer, respectively.
